# Porous Carbon Nanoflakes Doped with Boron Derived from Carbon Fabric Containing Polyester as Efficient Electrocatalysts for Green Hydrogen Production

**DOI:** 10.3390/polym18091107

**Published:** 2026-04-30

**Authors:** Syed Mohammed Hubaish, Mohammed Saad, Fadwa Eljack, Mira Chitt, Latofat Mahkamova, Kamel Eid

**Affiliations:** 1 Gas Processing Center (GPC), College of Engineering, Qatar University, Doha 2713, Qatar; sh2314847@student.qu.edu.qa (S.M.H.); m.saleh@qu.edu.qa (M.S.); 2 Department of Chemical Engineering, College of Engineering, Qatar University, P.O. Box 2713, Doha 2713, Qatar; fadwa.eljack@qu.edu.qa; 3 College of Engineering and Technology, University of Doha for Science and Technology, Doha 24449, Qatar; mira.chitt@udst.edu.qa; 4 Department of Organic Synthesis Technology, Tashkent Chemical-Technological Institute, Tashkent 100011, Uzbekistan; l.maxkamova@mail.ru

**Keywords:** porous carbon nanoflakes, green hydrogen, hydrogen evolution, electrocatalyst, water splitting

## Abstract

Developing Pt-free electrocatalysts is the main solution for reducing the intolerable cost of hydrogen production through the hydrogen evolution reaction (HER), while sustaining rare-earth elements. Thus, we have synthesized carbon nanoflakes derived from carbon cloth doped with controllable boron atoms (Bx/C), where x refers to boron atomic contents (x = 3.42, 5.04, 9.79, and 14.64 wt.%), driven by the impregnation of carbon cloth containing polyester (CC) in an aqueous solution of boric acid, followed by drying at 80 °C for 1 h and then calcination at 500 °C for 2 h under nitrogen. The method allows the conversion of one-dimensional CC to a two-dimensional flake-like structure, in situ enriched with B-C motifs as active sites for HER. The HER performance depends on interfacial interaction of boron with carbon, but B_1_/C (B = 3.42 wt %) was the optimum with a HER current of 370 mA/cm^2^ at −0.78 V, overpotential at 10 mA/cm^2^ (ƞ_HER@10_) of 372 mV, Tafel slope of 166 mV/dec, and stability for 60 h, besides a hydrogen production rate of 1.57 mol·g^−1^·h^−1^ of catalyst, due to endowing surface area, intermolecular charge transfer, and electrical conductivity. The data obtained may pave the way for designing heteroatom-integrated carbon from biomass for promoting low-cost HER.

## 1. Introduction

Hydrogen is considered to be either the fuel for the future or an energy carrier, owing to its massive energy per mass along with feasibility for hydrogen production via different methods from abundant resources, such as water, biomass, and CO_2_/CH_4_ [1,2,3]. Hydrogen production via water splitting (HER) is a green, efficient, and sustainable approach [4,5,6]. The HER usually requires metal-based cathodes, such as Pt-group catalysts [7], metal sulfides, and metal phosphides [8,9], which raise the cost of hydrogen production over 7.5 USD/1 kg [10]. Developing a Pt-free electrocatalyst, such as carbon nitride [11,12], metal-organic frameworks [13], and carbon-based catalysts [14,15] for HER, is one of the most efficient approaches not only for reducing the total HER cost but also preserving rare-earth elements and attaining sustainability [3,10,16,17]. Distinct from other carbonaceous materials, carbon cloth (i.e., fabric/cloth/textile) fibers are produced commercially at low cost using various methods from earth-abundant resources (i.e., coal, wood, peat, biomass, and synthetic polymers). They possess outstanding flexibility, surface area, strength-to-weight ratio, tailored porosity, electrical conductivity, and chemical durability in various electrolytes [18,19,20]. Also, the high porosity can accelerate mass transfer and promote interaction with the electrolyte, leading to maximize the utilization of active sites during the HER [21,22,23,24,25,26]. Thus, carbon cloths are usually used as substrates for various metals, oxides, and chalcogenides to be used as electrodes for various electrocatalytic applications, with rare reports on HER [27,28,29,30]. Carbon cloth materials were used as support for various metals, such as FeP [31,32], binary metals (i.e., MnCo) [33,34], S-rich MoS_2_ [35], Co rods [36], NiO@Ni [37], and Pt/CoS_2_ [38], for HER. For instance, nitrogen-doped carbonized cotton cloth formed by carbonization of cotton cloth followed by polyacrylonitrile treatment and annealing [39] showed an HER current of 100 mA/cm^2^ at −0.40 V, an η_HER@10_ of 233 mV, and stability for 15 h in 1 M KOH. Porous graphite carbon@oxidized carbon cloth co-doped with N, P, and O enhances the HER with η_HER@10_ of 446 mV and Tafel slope 154 mV/dec in 1M KOH [40]. CC supported N-doped graphen (N-GCSs) displayed Tafel slope of 198 mV/dec and η_HER@10_ −0.320 V_RHE_ in H_2_SO_4_ electrolyte [41]. Notably, the effect of heteroatom doping (i.e., B, O, F, and P) with CC on the HER without metal nanocrystals is still ambiguous, and the HER activity is still not efficient enough to replace Pt-based catalysts. Also, the role of dopants in the HER is still in a huge controversy, and the doping method increases the complexity of the fabrication process, which greatly limits its practical utilization.

To this end, carbon nanoflakes derived from carbon cloth were doped with controllable boron atoms (B_1_/C, B_2_/C, B_3_/C, and B_4_/C) with boron contents of 3.42 wt.%, 5.04 wt.%, 9.79 wt.%, and 14.64 wt.%, respectively. The fabrication approach entails the direct soaking of carbon cloth in a solution of boric acid for 5 min, then drying at 80 °C for 1 h, and finally calcinating at 500 °C for 2 h under nitrogen. The shape, composition, and crystallinity of the formed materials were investigated using various tools like SEM, EDX mapping, XRD, XPS, and Raman spectroscopy. The electrolytic activities of thus-formed materials were tested toward the HER in H_2_SO_4_ electrolytes, which depend on the boron content. B_1_/C showed the highest HER activity with a HER current of 370 mA/cm^2^ at −0.78 V, an ƞ_HER@10_ of 372 mV, a Tafel slope of 166 mV/dec, and stability for 24 h besides a hydrogen production rate of 1.57 mol·g^−1^·h^−1^. Unlike previous reports, this study provides a simple (i.e., soaking and annealing), fast (i.e., ≤4 h), and scalable approach (i.e., up to several grams per run) for the synthesis of metal-free cathodes for the HER without the need for multiple-step reactions or hazardous chemicals that can meet sustainability requirements. Also, the integrations of boron atoms with carbon led to interfacial interaction that enhanced electrical conductivity and promoted the interaction of electrolyte-electrode during HER.

## 2. Experimental and Chemicals

### 2.1. Chemicals

Boric acid (H_3_BO_3_ ≥ 99.5%), ethanol solution (CH_3_CH_2_OH, 99.8%), and a commercial Pt/C catalyst (20 wt. % Pt) were purchased from Sigma-Aldrich Chemie GmbH, Eschenstr. 5, 82024 Taufkirchen, Germany. Twill weave carbon fiber-polyester (CC) was purchased from Qingdao Aerolite Technology Co., Ltd., Qingdao, Shandong 266000, China.

### 2.2. Preparation of Boron Doped Carbon (B_x_/C)

For the synthesis of B_1_/C, CC (3 cm × 3 cm) was initially cleaned for three consecutive cycles in a solution of acetone/ethanol (1/1 VV ratio) under sonication for 30 min. The as-cleaned CC was directly soaked in a 3 mL solution of H_3_BO_3_ (0.2 M) for 5 min till complete adsorption. It was then dried at 80 °C for 1 h in an oven under air, followed by calcination at 500 °C for 2 h under nitrogen [33]. The samples B_2_/C, B_3_/C, and B_4_/C were prepared by the same method but using 0.3, 0.6, and 0.85 M of H_3_BO_3_, respectively.

### 2.3. Materials Characterization

The scanning electron microscope (SEM; Hitachi, Tokyo, Japan), equipped with an energy-dispersive spectrometer (EDX), was used for imaging and composition of the thus-formed materials. The X-ray photoelectron spectroscopy (XPS) Thermo ESCALAB 250 spectrometer was used for recording the valence states. X-ray diffraction (SRD) patterns from the X’Pert-Pro MPD (PANalytical Co., Almelo, The Netherlands) were used for collecting the diffraction patterns and crystalline structure. The Raman spectra were recorded on a micro-Raman spectrometer (Renishaw InVia Confocal Raman Microscope, Renishaw plc, Wotton-under-Edge (Gloucestershire), UK) with a laser beam at λ = 532 nm.

### 2.4. The HER

For the HER test, we used a three-electrode cell of the graphitic bar (5 mm) (working electrode) and Ag/AgCl (reference electrode). All the electrochemical tests were conducted on a Gamry potentiostat (Gamry 3000). The Bx/C (2 mg each) was dissolved in a solution of 1 mL of ethanol/H_2_O/Nafion (3/1/0.1 *v*/*v*/*v*) and then drop-casted (10 µL each) on a carbon cloth sheet (2 cm × 2 cm) and left to dry under air at 80 °C for 1 h before usage. All recorded currents were corrected against the Ohmic potential drop and then normalized to the geometric area of the working electrodes. All the Ag/AgCl potentials were normalized to the reversible hydrogen electrode (RHE) using this equation (Equation (1)).

E_RHE_ = E_Ag/AgCl_ + 0.197 V + 0.059 X pH (1)

The obtained H_2_ from the HER was collected using gas bags (multi-layer foil barrier, low permeability HedeTech Co., Ltd., Dalian, China) and then estimated using a gas chromatograph (Shiweipx GC-7806 (Beijing Shiweipuxin Technology Co., Ltd., Beijing, China), TCD detector, N_2_ as the carrier gas, and a 5 Å molecular sieve column).

## 3. Results and Discussions

**Scheme 1:**
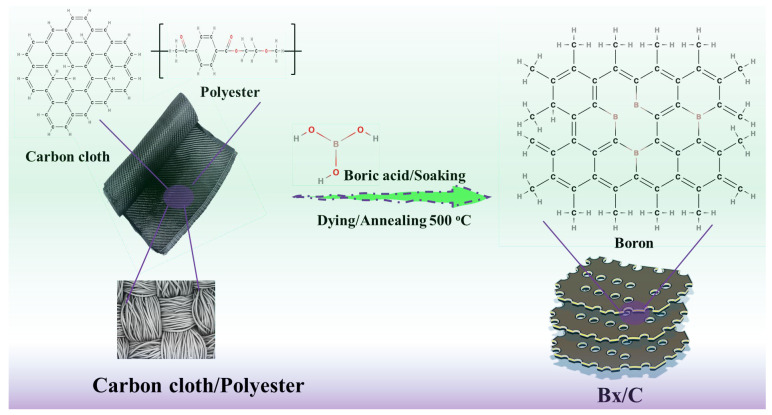
Illustration of the formation process of B_x_/C.

Scheme 1 shows the preparation method for the thus-formed Bx/C, which comprises soaking CC in an aqueous solution of boric acid (H_3_BO_3_) and then drying and calcination at 500 °C under nitrogen. During soaking, the monobasic Lewis H_3_BO_3_ acid is easily adsorbed coherently by CC, owing to high porosity with interconnected pores that act as capillary channels. Upon annealing under nitrogen, H_3_BO_3_ decomposes in a multi-step dehydration process, ultimately forming metaboric acid (HBO_2_) and water vapor at 100–150 °C, and it then dehydrates metaboric acid to form tetraboric acid (H_2_B_4_O_7_) at 150–300 °C, followed by the formation of boron trioxide (B_2_O_3_) at >300 °C [42]. Following that, a meeting occurs, and B_2_O_3_ forms a molten film over the carbon, and the interaction occurs chemically with the carbon surface and any O_2_-containing functional groups (like hydroxyl or carboxyl groups) of CC, and the doping takes place via diffusion of boron atoms into the CC matrix above 500 °C via boron replacing carbon atoms in the lattice or occupying spaces between layers [43]. Notably, the CC is thermally degraded to form carbonaceous biochar at high temperatures due to the high polyester resin, leading to the conversion of the CC fiber into carbon flake layers. Figure S1a shows the CC used as a precursor for the preparation process, which is commercially available and easily prepared at low cost. This CC, after impregnation and annealing, turned to black power of Bx/C (Figure S2b), which confirms the high mass production and scalability of the presented method. This is evident in the complete conversion of the CC to powder with no significant loss.

**Figure 1:**
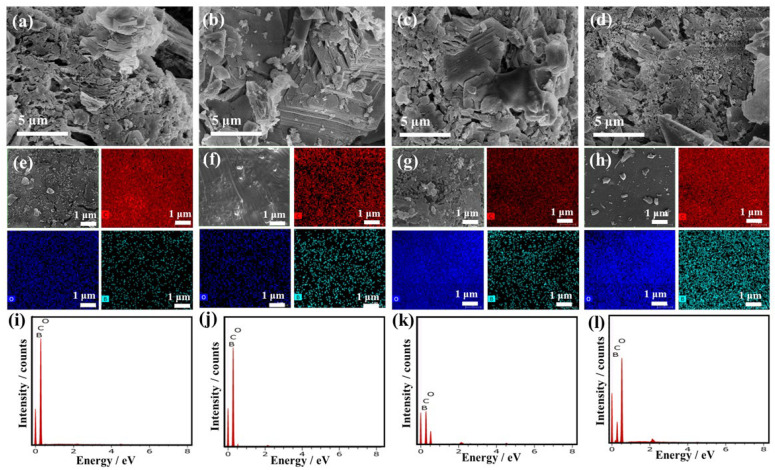
The SEM images of B1/C (**a**), B2/C (**b**), B3/C (**c**), and B4/C (**d**). The elemental mapping images (**e**–**h**) and EDX spectra (**i**–**l**) of B1/C, B2/C, B3/C, and B4/C, respectively.

This is further seen in the SEM analysis used to investigate the morphology and composition of the thus-formed materials. SEM images of the as-synthesized Bx/C materials display the successful formation of a carbon flake-like structure with high porosity (Figure 1a–d). Varying the doping content led to variations in the layers’ structure and porosity, attributed to the in situ released gas from the decomposition of either H_3_BO_3_ (i.e., H_2_O) or polyester (i.e., CO and CO_2_) [44] during calcination. The EDX elemental mapping images show the coherent distribution of C with B and O atoms (Figure 1e–h) due to the ease of molten B_2_O_3_ melting within carbon. The EDX spectra show the co-existence of B-atoms with atomic content of 3.42, 5.04, 9.79, and 14.64 wt.% in B_1_/C, B_2_/C, B_3_/C, and B_4_/C, respectively (Figure 1i–l; Table 1). The resolved oxygen is plausible due to oxygen-rich groups in the polyester resin of CC, which is crucial for promoting the hydrophilicity and wettability during HER, which can endow the interaction between the electrolyte and the electrode [33].

**Figure 2:**
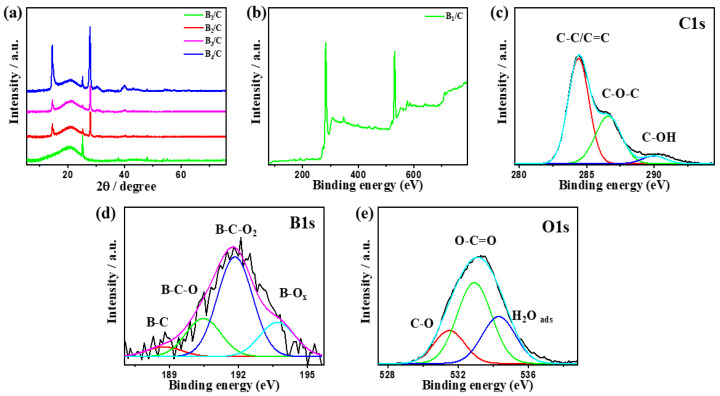
XRD spectra of thus formed Bx/C materials (**a**). The XPS full scan of B1/C (**b**) and its high-resolution XPS spectra of C1s (**c**), B1s (**d**), and O1s (**e**).

We have conducted XRD analysis to investigate the crystallinity and interaction of B atoms with C atoms in the thus-synthesized Bx/C materials (Figure 2a). The XRD patterns of B_1_/C, B_2_/C, B_3_/C, and B_4_/C indicate structural evolution with increasing boron content, with B_4_/C showing the most distinct boron-related diffraction features (Figure 2a). A broad reflection in the 24–26° region is attributable to the (002) plane of graphitic carbon that is indexed to graphite (PDF/JCPDS 41-1487) [45]. In boron-rich samples, the carbon-related band becomes broadened and slightly perturbed, consistent with boron-induced lattice distortion, reduced stacking regularity, and increased defect density in the carbon matrix. Such broadening, reflected by the larger apparent FWHM of the carbon-related envelope, indicates a reduction in coherent scattering length along the stacking direction and therefore a decrease in structural order. In parallel, additional reflections emerge or become more intense in the approximate ranges of 14.9°, 21.7°, 28.8°, 31.4°, and 41.8°. These features can be reasonably assigned to boron-containing phases, including boron carbide domains indexed against B_4_C (PDF/JCPDS 35-0798) and oxygen-containing boron species or boron–carbon–oxygen environments referenced against B_2_O_3_-related patterns (PDF/JCPDS 06-0297) [46]. The increase in intensity of these peaks from B_1_/C to B_4_/C supports the conclusion that higher boron loading promotes the development of B–C and B–O coordinated structural motifs (Figure 2a). Using Bragg’s law, d=λ/(2sin θ) with Cu Kα radiation (λ=1.5406 Å), the principal peaks extracted from the uploaded figure give approximate d-spacings of 5.921, 4.098, 3.093, 2.848, and 2.159 Å for the reflections centered at 14.95°, 21.67°, 28.84°, 31.39°, and 41.81°, respectively, which are consistent with the coexistence of disordered graphitic carbon and boron-containing crystalline subdomains. The apparent crystallite sizes were estimated from the Scherrer equation, D=Kλ/(βcos θ), using K=0.9, which gave approximate coherent domain sizes of about 5.20, 5.0, 4.7, 4.30, and 4.27 nm for B_1_/C, B_2_/C, B_3_/C, and B_4_/C, respectively. Notably, increasing B-content causes significant lattice perturbation, broader defect-associated carbon reflections, and stronger boron-derived crystalline signatures, with B_4_/C exhibiting the highest degree of boron-related phase development.

The valence states and surface distribution of B atoms within C atoms in B1/C were demonstrated using the XPS analysis. The full-scan XPS spectra show the valence states attributed to C1s, O1s, and B1s in the B1/C sample (Figure 2b). The high-resolution XPS spectra of B1s show the valence states assigned to B-C-O_2_ as the main peak, along with weak phases of B-Ox, B-CO, and B-C (Figure 2d), implying the successful integration of B within carbon atoms [45,46,47]. These different B-C functionalities, with dissimilar electronegativities and electron pairs, can act as active sites during HER, leading to enhanced activity and durability [48,49]. The C1s spectra show the valence state assigned to C-C/C=C of graphite carbon as the main phase, along with other phases of C-O-C and C-OH. The high oxygen content is due to the original oxygen in polyester CC that is used as a precursor for the preparation of B_1_/C. The oxygen moieties are crucial for promoting wettability and interactions between Bx/C and the electrolyte during HER. The C-C/C=C peak was slightly shifted toward lower binding energies compared with the reference peak position, owing to boosting the electron density on C atoms after B-doping due to the charge transfer between B and C. The lower electronegativity and higher work function of B atoms than carbon led to electron transfer via contact to equilibrate their Fermi levels, resulting in an increase in the electron density of carbon and reducing their binding energy [48,49]. The binding energy shift can improve electrical conductivity, which is superior for greater interfacial interaction and accelerated electron transfer, leading to accelerated reduction of protons (H^+^) to H_2_ from water in the electrolyte during the HER, which is important for the Volmer step (H^+^ adsorption) and Heyrovsky/Tafel steps (H_2_ desorption) [48].

**Figure 3:**
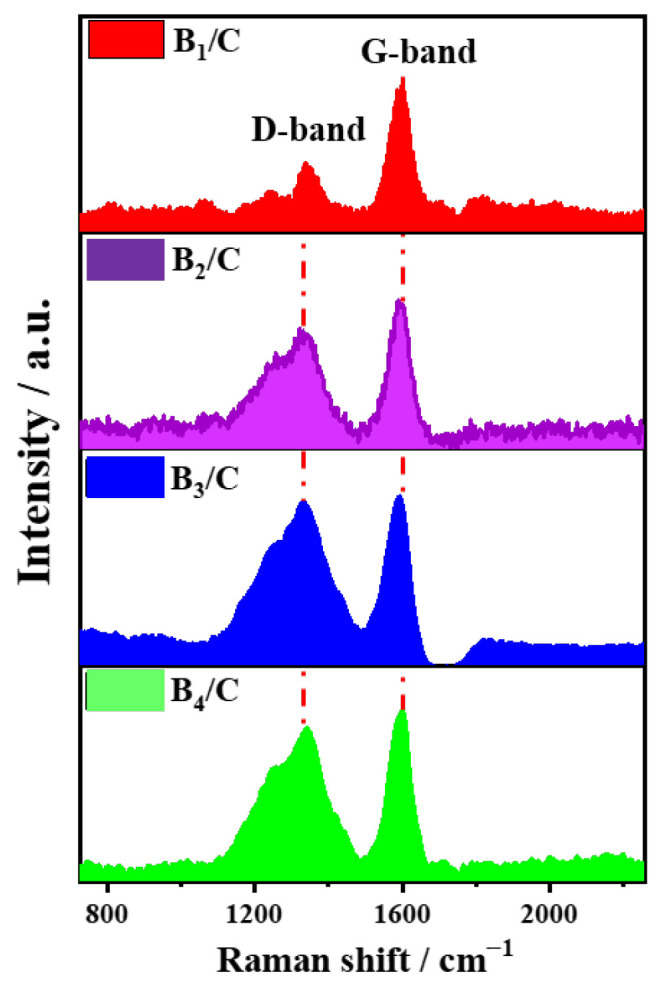
Raman analysis of Bx/C materials.

The effects of B-atoms on the disorder in sp2 C-atoms and the interfacial interaction in Bx/C were investigated using Raman spectroscopy (Figure 3). All Bx/C showed two peaks attributed to the crystalline (D-band) at 1351 cm^−1^ and the graphite carbon (G-band) at 1590 cm^−1^, which are the main features of graphitic carbon formed by calcination. The effect of B-atoms is shown in the dissimilar ratio of the D-to-G band peak intensities (ID/IG) that were estimated to be around 0.399, 0.740, 0.923, and 0.895 for B_1_/C, B_2_/C, B_3_/C, and B_4_/C, respectively. The lower (ID/IG) ratio in B_1_/C could serve as an indication of better graphitization degree, with minimal defects, as seen in the higher intensity of the G-band and reducing the size of sp^2^ domains [38,46]. This is seen in the lower D-band peak intensity with less broadening of B_1_/C, more than its counterparts, Bx/C, which are indications of low crystal lattice edge defects and higher “ordered” graphitic domains, originating from lower content of B-doping [45]. This is also seen in the increasing D-band peak intensity with increasing broadening, which is assigned to decreasing the crystallite size. B-atoms are interrupting the long-range order of the C-atoms, turning a once-pristine structure into a network of very small, boron-interrupted graphitic clusters. This is seen in increasing D-band peak intensity that implies a higher concentration of substitutional dopants and vacancies, along with intermolecular charge transfer, in line with the high ratio of ID/IG bands, as usually observed in carbon materials functionalized with metal/non-metal atoms [50]. The low content of B-atoms can act as a p-type dopant, leading to interfacial charge transfer, where boron (an electron acceptor) withdraws electrons from the carbon lattice, and creating a hole. Meanwhile, the D-band intensity is non-linearly related to charge transfer; it typically reaches a maximum when the Fermi level approaches half of the laser excitation energy.

**Figure 4:**
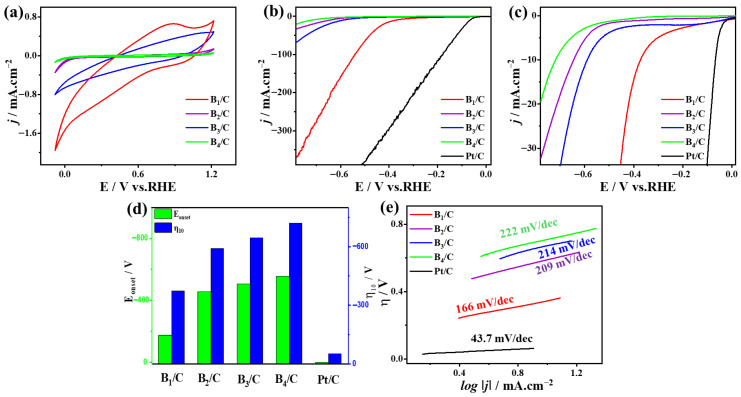
(**a**) CV tests, (**b**) LSV tests, (**c**) LSV curves magnified, and comparison of ƞ_10_ and E_Onset_, (**d**) Tafel plots, and (**e**) tested in 0.5 M H_2_SO_4_ compared with Pt/C catalyst.

The electrocatalytic HER activity and stability of Bx/C materials were investigated as a function of B atoms using various tests in 0.5 M H_2_SO_4_ electrolytes. Cyclic voltammetry (CV) tests were initially tested for Bx/C at 200 mV/s for 100 cycles while nitrogen was purging to ensure cleaning and removing impurities from the electrode surfaces that can be seen in stabilized current density. Then, the CV curves of Bx/C were recorded at 50 mV/s, which showed the semi-rectangle shape assigned to voltammogram features of carbon with a high capacitance effect (Figure 4a) [51]. Notably, B_1_/C showed a higher capacitance current and an obvious underpotential adsorption/desorption area compared with other Bx/C, which serves as evidence for a higher electrochemically active surface area (ECSA) and better hydrogen generation. Thus, the CV curves were tested at different scan rates ranging from 25 mV/s to 250 mV/s on Bx/C and all materials, which revealed an increasing HER current with boosting the scan rates on all materials (Figure S2), which is an indication of the diffusion-controlled process. Plotting the HER current versus the square roots of scan rates and scan rates showed a linear relationship. B_1_/C had a superior slope value among all tested materials, which serves as an indication for promoting the kinetics and charge diffusion during HER (Figure S2) [33]. The electrochemically active surface areas (ECSAs) of B_1_/C, B_2_/C, B_3_/C, and B_4_/C were about 45.5, 9.1, 26.75, and 3.5 cm^2^, respectively. The ECSA of Pt/C was about 71 m^2^/g, as estimated from the coulombic charge obtained from integrating the hydrogen underpotential deposition area. The higher normalized ECSA per catalyst weight of B_1_/C (4.2 × 10^5^ cm^2^/g) that comparable to Pt/C (7.1 × 10^5^ cm^2^/g) indicates the rich active sites that are crucial for the enhancement of HER.

Linear sweep voltammetry (LSV) was tested at 2 mV/s on all materials compared with commercial Pt/C, which all demonstrate the features of HER, including high cathodic current in the negative potential direction. The HER current increased with the increasing applied negative potential originating from water splitting and the consequent release of hydrogen gas (Figure 4b). The HER current density of B_1_/C was higher than that of B_3_/C, B_2_/C, and B_4_/C and was closest to that for Pt/C. Primarily, the obtained HER current on B_1_/C (370 mA/cm^2^) was superior to B_3_/C (70 mA/cm^2^), B_2_/C (33 mA/cm^2^), and B_4_/C (20 mA/cm^2^). Notably, B_1_/C can deliver a higher current at any applied voltage point, which indicates maximized usage of the B atom in B_1_/C than its counterparts (Figure 4c and Table S1). The higher HER activity of B_1_/C is shown in its ability to consume lower overpotential at 10 mA/cm^2^ (ƞ_HER@10_) (−372 mV) than those of B_3_/C, B_2_/C, and B_4_/C (Figure 4d), as also seen in the lower onset potential (E_Onset_) (Figure 4c and Table S1). The lower ƞHER@10 and E_Onset_ are obvious evidence for the accelerated HER kinetics on B_1_/C and Cu/h-CNFs. This is also seen in the lower Tafel slope on B_1_/C (166 mV/dec) than in B_3_/C, B_2_/C, and B_4_/C, which serve as additional proof for the quickest HER kinetics on B_1_/C (Figure 4e). The noticed larger value of Tafel slopes on Bx/C probably originated from strong adsorption of protons on the monolayer coverage on the carbon surface during HER [52]. Based on the estimated Tafel slope value, it indicates sluggish proton adsorption kinetics, and the HER mechanism follows the Volmer-Heyrovsky pathways, with the Volmer step as the rate-determining step [48], which entails electrochemical H-adsorption and, eventually, electrochemical H_2_-desorption of hydrogen. The H_2_ production rates were estimated to be 1.57, 0.16, 0.075, and 0.033 mol·g^−1^·h^−1^ @ 650 mV on B_1_/C, B_2_/C, B_3_/C, and B_4_/C, respectively (Table S1). The higher H_2_ production rate on B_1_/C is due to better utilization of the B-C active sites during HER [48,49]. Notably, undoped CC displayed an inferior HER activity (Figure S3).

**Figure 5:**
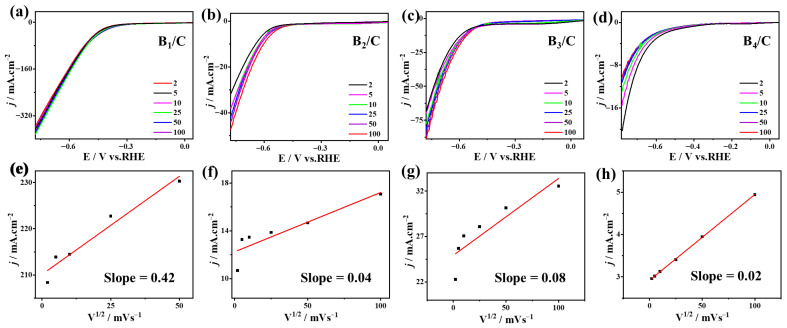
LSV tested at different scan rates (**a**–**d**) and their current versus the square roots of scan rates (**e**–**h**) on the as-made Bx/C materials.

The LSV curves were tested at different scan rates ranging from 2 mV/s to 100 mV/s on Bx/C. The LSV of B_1_/C showed stable current with increasing scan rates, implying the stabilized HER, while other Bx/C displayed fluctuation in the HER current at different scan rates (Figure 5a–d). The liner relationship obtained from plotting current density versus the square root of scan rates (v^1/2^) on all Bx/C materials implies a diffusion-controlled process in the low voltage area. Moreover, the lines’ slope of B_1_/C was greater than that of its counterparts (Figure 5e–h). The higher slope line is an indication of better diffusion and quicker HER kinetics.

**Figure 6:**
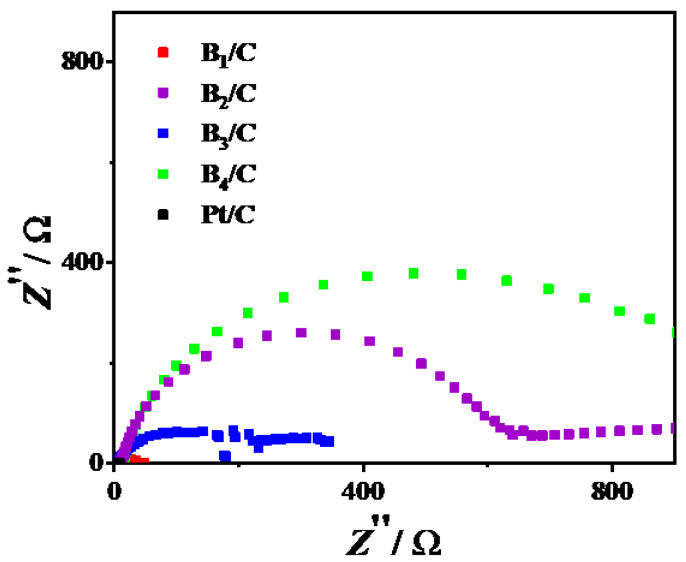
EIS Nyquist plots of Bx/C electrocatalysts tested in 0.5 M H_2_SO_4_ compared with Pt/C catalyst at −0.6 V.

To gain more insights into the effect of B atoms on HER activity, EIS Nyquist plots were recorded for Bx/C compared with Pt/C, which all displayed semicircle lines but with a lower diameter in the order of B_1_C < B_3_/C < B_2_/C < B_4_/C (Figure 6). The lower line diameter of B_1_/C is closer to Pt/C. The lower diameter B_1_/C is an indication of their lower charge-transfer resistance than other Bx/C. The EIS fitting conducted using a Voigt electrical equivalent circuit showed smaller electrolyte resistance (*Rs)* and charge transfer resistance (*R*_ct_), along with higher constant phase elements (CPE) of B_1_/C than those of B_3_/C, B_2_/C, and B_4_/C (Table 2) [33]. The values of B_1_/C were closer to Pt/C, which indicates higher electrical conductivity and better electrolyte-electrode interaction on B_1_/C, in line with the higher intermolecular charge transfer in the Raman and XPS analysis. The electron transfer between B atoms and C atoms, along with higher electron deficiency, altering their d-band centers, led to optimizing H_2_-adsorption/desorption strength during HER.

The HER chronoamperometry tests of Bx/C were measured for 60 h, which demonstrate better stability of B_1_/C than its counterparts, as seen in the lower current attenuation and higher current retention after 16 h (Figure S4). This is due to the great synergy of B and C in Bx/C and the homogeneous distribution of B atoms within the carbon skeleton structure, which prevents B-detachment during HER. It is noteworthy that B_1_/C showed better activity, including higher current density with a lower overpotential, than that of other C and graphene doped with B alone or other heteroatoms such as 5% F/BCN [48], B SuG, [49], BCN-3 [53] (Table 3), in addition to the simplicity, scalability, and green of the preparation method of B_1_/C. The superior activity of B_1_/C is plausibly originated from the homogenous distribution of rich B-C active sites, their interfacial interaction, the two-dimensional structure enriched with oxygenated functional groups, and intermolecular charge transfer. These merits led to promoting interaction with the electrolyte, enhanced mass transfer, and improved water splitting under low potential [33].

## 4. Conclusions

This work showed simple, one-pot, and green synthesis of porous carbon flakes derived from carbon cloth with controllable B dopants (Bx), where x is the atomic content of B atoms (x = 3.42, 5.04, 9.79, and 14.64 wt.%). The synthesis approach entails the soaking of carbon cloth in an aqueous solution of boric acid with different contents, followed by drying at 80 °C for 1 h and then annealing at 500 °C for 2 h under nitrogen. The materials were analyzed by various characterization techniques such as SEM, EDX mapping, XPS, XRD, and Raman spectra. The HER activity and stability of Bx/C were tested in H_2_SO_4_ electrolytes as a function of boron loading and were compared with Pt/C. The HER performance and stability were in the order of B_1_C > B_3_/C > B_2_/C > B_4_/C. Notably, B_1_/C (B = 3.42 wt%) was the best with a HER current of 370 mA/cm^2^ at −0.78 V, an overpotential at 10 mA/cm^2^ (ƞ_HER@10_) of 372 mV, a lower Tafel slope of 166 mV/dec, and stability for 60 h beside a hydrogen production rate of 1.57 mol·g^−1^·h^−1^ @ 650 mV. The obtained data displayed that the interfacial interaction of boron with carbon plays a key role in promoting the HER activity at low cost. Although the simplicity and scalability of the thus-reported B1/C catalyst are still far beyond practical applications that require high current density in the A/cm^2^ range with stability for a few weeks. This issue could be addressed via the insertion of Pt-based as an atomic dopant or a single atom with very low loading to reduce the energy barrier and maximize current density. This work paves the way for designing scalable, low-cost, and eco-friendly other heteroatoms integrated carbon-derived form polymeric cloth materials for sustainable hydrogen production across a wide pH range.

## Figures and Tables

**Table 1:** Elemental composition of B_x_/C catalysts by the EDX technique.

Catalyst	B	C	O
B_1_/C	3.42 ± 0.031	94.56 ± 0.95	2.02 ± 0.011
B_2_/C	5.04 ± 0.042	88.13 ± 0.9	6.84 ± 0.053
B_3_/C	9.79 ± 0.084	59.89 ± 0.602	24.34 ± 0.23
B_4_/C	14.64 ± 0.13	58.14 ± 0.55	27.22 ± 0.22

**Table 2:** EIS fitting data of Bx/C relative to Pt/C.

Catalyst	*R*_s_ (Ω)	*R*_ct_ (Ω)	CPE (μF.s^(1−*a*)^)	*a*
B_1_/C	5.45 ± 0.065	27.58 ± 0.26	73.23 ± 5.8	0.887 ± 0.008
B_2_/C	6.35 ± 0.095	640.3 ± 13.21	44.24 ± 1.06	0.872 ± 0.003
B_3_/C	6.135 ± 0.057	216.6 ± 2.448	81.16 ± 2.77	0.769 ± 0.004
B_4_/C	7.994 ± 0.082	985.8 ± 6.134	21.76 ± 0.6	0.7420 ± 0.003
Pt/C	4.9 ± 0.21	2.3 ± 0.1	117.65 ± 3.211	0.91 ± 0.011

**Table 3:** Comparison of the HER activity of our newly designed B1/C relative to other catalysts.

Catalyst	Preparation Method	Electrolyte	Overpotential (mV) @ 10 mA cm^−2^	Tefal Slope (mV/dec)	Ref.
B_1_/C	Soaking/drying and annealing at 550 °C	0.5 H_2_SO_4_	372	166	OUR WORK
5% F/BCN	Pyrolysis-assisted solution-phase hybridization method	0.5 H_2_SO_4_	437	153	[1]
B SuG	Hummer’s method, reduction and reflux	0.5 H_2_SO_4_	~445	130	[2]
FLBP@BG	Exfoliation of BP/Oxidation of GO, annealing/sonication	0.5 H_2_SO_4_	385.9	110	[3]
g-C3N4/BN@Ct	Thermal decomposition polymerization and ultrasonication	0.5 H_2_SO_4_	520	150	[4]
O-SiBCN/rGO	Hummer’s method, vacuum-assisted impregnation, and annealing	0.5 H_2_SO_4_	590	127	[5]
BCN-3	Ultrasonication mixing and annealing	0.5 H_2_SO_4_	487	195	[6]

## Data Availability

The raw data supporting the conclusions of this article will be made available by the authors on request.

## Supplementary Materials

The following supporting information can be downloaded at: https://www.mdpi.com/article/10.3390/polym18091107/s1. Figure S1: Photograph of commercially pusrshed twill weave carbon cloth fiber containing polyester (CC) (a) and (b) B1/C powder obtained after impregnation and annealing of CC. powder. Figure S2: CV curves at different scan rates (a–d), current versus square roots of scan rates (e–h), and current versus scan rates (i–l) on B1/C, B2/C, B3/C, and B4/C. Figure S3. LSV of the control sample tested in 0.5 M H_2_SO_4_. Figure S4. Chronoamperometry tests of Bx/C compared with Pt/C measured at −0.6 V. Table S1. Electrocatalytic HER activities of B_x_/C catalysts in 0.5 M H_2_SO_4_ solution.
