# Supplementary material for: Porous Carbon Nanoflakes Doped with Boron Derived from Carbon Fabric Containing Polyester as Efficient Electrocatalysts for Green Hydrogen Production
Source: Polymers (Basel). 2026 Apr 30;18(9):1107. doi: 10.3390/polym18091107 (PMC13165666; doi:10.3390/polym18091107)
Supplement: Supplementary file 1 [file polymers-18-01107-s001.zip › polymers-4279832-supplementary.pdf]

Supporting information file

Porous Carbon Nanoflakes doped with Boron Derived from Carbon Fabric Containing Polyester as Efficient Electrocatalysts for Green Hydrogen Production

Syed Mohammed Hubaish [1], Mohammed Saad[1,2], Fadwa Eljack[2], Mira Chitt[3], Latofat Mahkamova[4], Kamel Eid[2,*]

1 Gas Processing Center (GPC), College of Engineering, Qatar University, Doha, 2713, Qatar; sh2314847@student.qu.edu.qa (S.M.H.),(kamel.eid@qu.edu.qa (K.E.)
2 Department of Chemical Engineering, College of Engineering, Qatar University, P.O. Box 2713, Doha 2713, Qatar; m.saleh@qu.edu.qa (M.S.); Fadwa.Eljack@qu.edu.qa (F.E.),
3 College of Engineering and Technology, University of Doha for Science and Technology, Doha 24449, Qatar; e Mira.chitt@udst.edu.qa (M.C.)
4 Tashkent Chemical-Technological Institute, Tashkent 100011, Uzbekistan; l.maxkamova@mail.ru (L.M)
* Correspondence: kamel.eid@qu.edu.qa; Tel.: (+974) 4403-4378)

Electrochemical HER calculations

- **The electrochemically active surface area ECSA (cm²)**

It's calculated by calculating the C_{dl} which equal to the slope of the measured average current density at 0.3 V versus different scan rate.

$$ECSA = C_{dl} / C_s$$

Where, C is the measured double-layer capacitance (F), C_s is the specific capacitance of a flat surface.

For carbon-based catalysts, the specific capacitance, C_s = 0.04 $mFcm^{-2}$. then normalized the loading of catalyst weight.

- **Hydrogen rate production**

The hydrogen production rate normalized to catalyst mass, the following equation was used:

$$H_2 \text{ rate} = (I \times 3600) / (2F \times m_{cat})$$

where, m_{cat} is the catalyst loading on the electrode (g), and the factor 3600 converts seconds to hours, giving the hydrogen evolution rate in mol g^{-1} h^{-1}.

- **TOF Calculation Based on Electrochemically Active Surface Area (ECSA)**

The number of electrochemically accessible active sites (n_{sites}) estimated by the active surface area (ECSA).

$$n_{sites}=\mathrm{ECSA}\times\Gamma$$

where, Γ is the surface site density, assumed to be 2×10^{-9} mol cm^{-2} for HER catalysts.

The intrinsic turnover frequency was then calculated as:

$$\mathrm{TOF} = \mathrm{I} / (2\mathrm{F}\times n_{sites})$$

where, I is the current at defined potential, and F is the faradic constant 96485 C mol^{-1}

- **Mass Activity**

Mass activity represents the current normalized to catalyst mass:

$$Mass\ \mathrm{Activity} = \frac{I}{m_{cat}}$$

Where, I is the measured current (A) and m_{cat} catalyst mass on electrode (g).

- **Specific Activity**

Specific activity represents the current normalized to **electrochemically active surface area (ECSA):**

$$Specific\ Activity = \frac{I}{ECSA}$$

Table S1. Electrocatalytic HER activities of B_x/C catalysts in 0.5 M H_2SO_4 solution.

Catalyst	ECSA (cm^2)	ECSA (cm^2/g) x 10^5	Onset / mV	Overpotential /mV	TAFEL mV/dec	TOF / s^{-1} @ 650mV	H_2 rate / mol g^{-1} h^{-1} @ 650mV	Mass activity (A g^{-1})	Specific activity (A m^{-2})
B_1/C	45.5	4.2	-175	372	166	1.53E-3	1.57	84.3	**5.92**
B_2/C	26.75	2.5	-456	589	214	2.66E-4	0.16	8.6	**1.03**
B_3/C	9.1	0.85	-506	645	209	3.66E-4	0.075	4.03	**1.42**

B_4/C	3.5	0.32	-556	719	222	4.15E-4	0.033	1.76	**1.60**
Pt/C		7.1	0	51	43.7	1.37	7.02	-	-

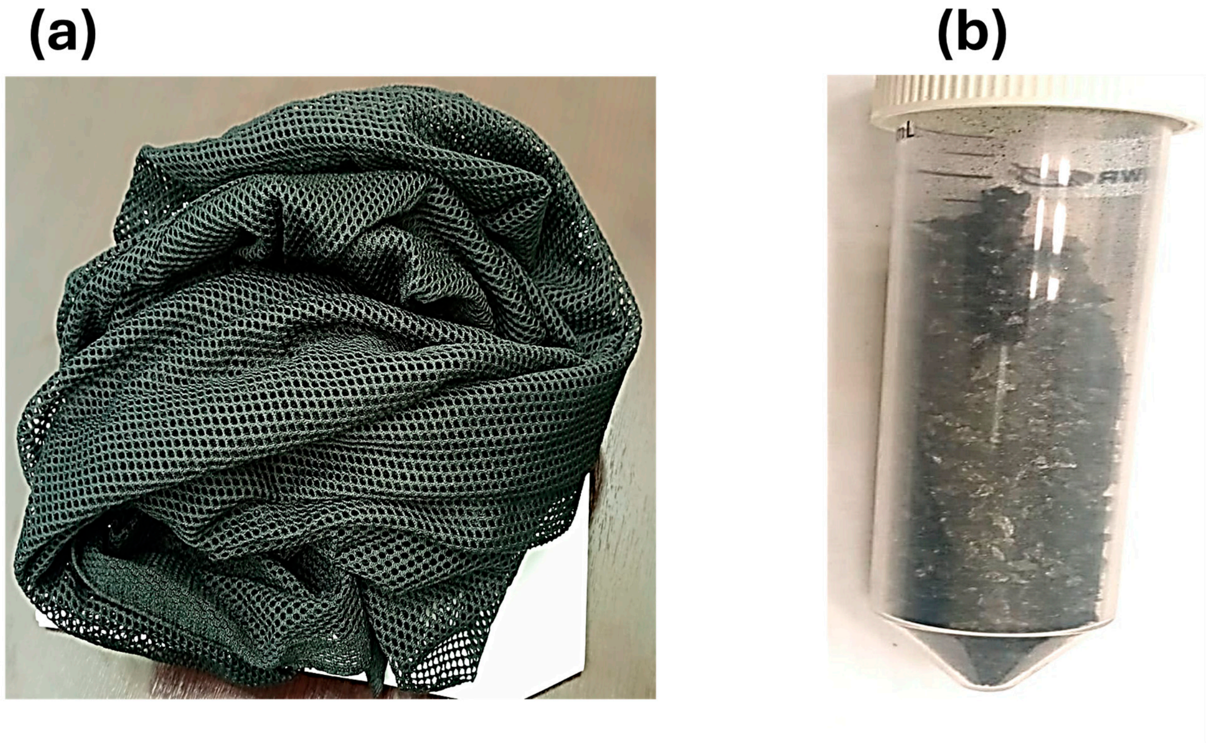

Figure S1 Photograph of commercially pusrshed twill weave carbon cloth fiber containing polyester (CC) (a) and (b) B1/C powder obtained after impregnation and annealing of CC. powder.

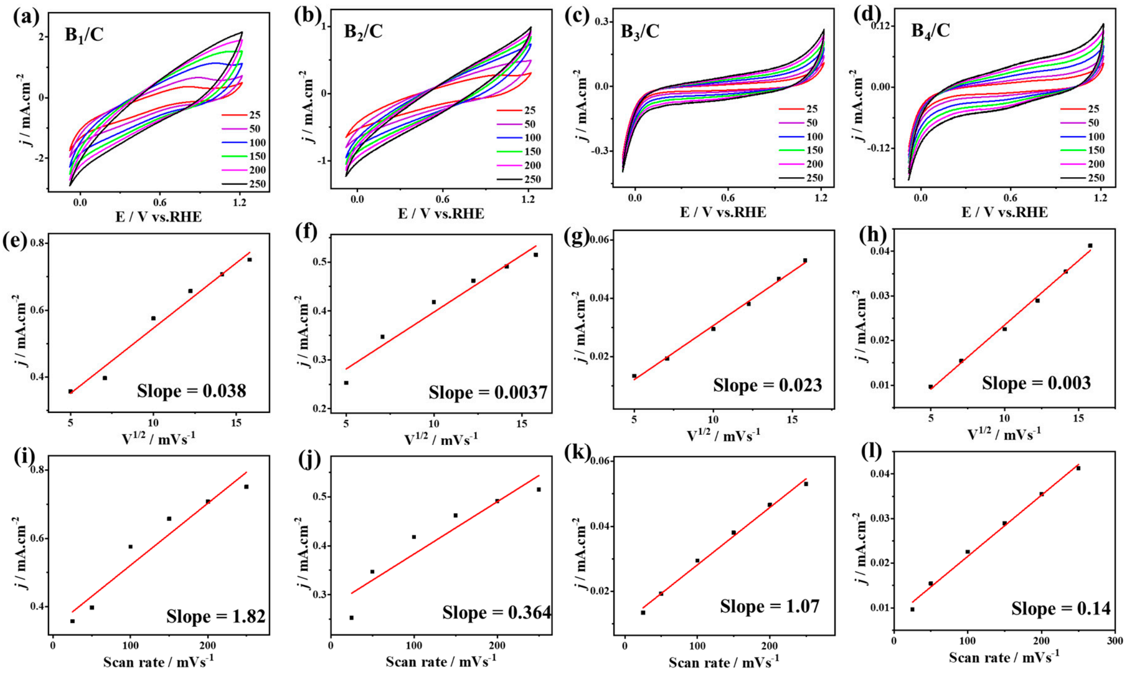

Figure S2. CV curves at differ scan rates (a-d), current versus square roots of scan rates (e-h), and current versus scan rates (i-l) on B_1/C, B_2/C, B_3/C, and B_4/C.

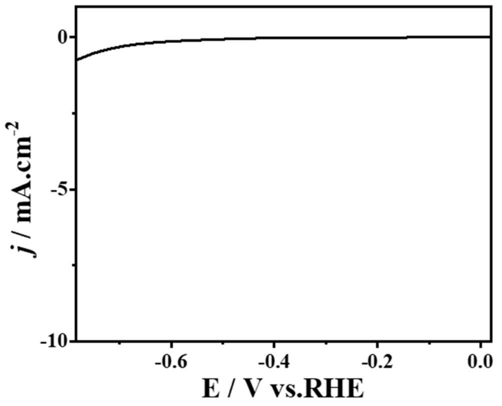

Figure S3. LSV of the control sampel tested in 0.5 M H_2SO_4.

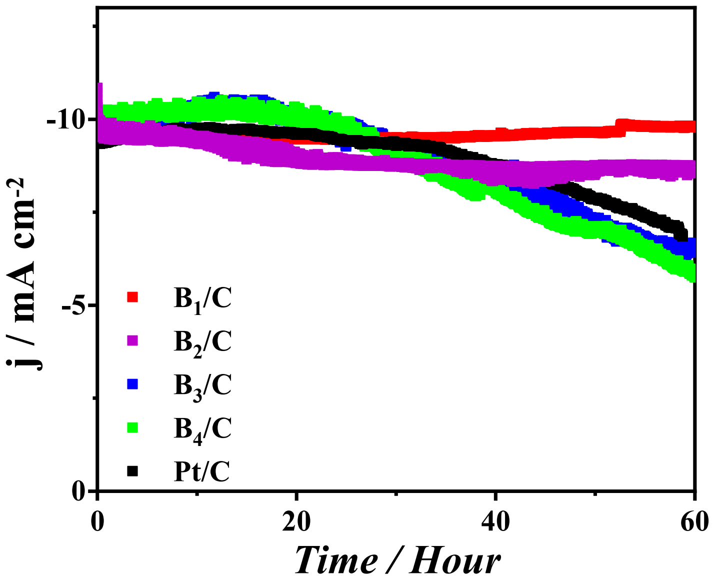

Figure S4. Chronoamperometry tests of Bx/C compared with Pt/C measured at -0.6V.
